# Intra-tropical movements as a beneficial strategy for Palearctic migratory birds

**DOI:** 10.1098/rsos.171675

**Published:** 2018-01-03

**Authors:** Jaroslav Koleček, Steffen Hahn, Tamara Emmenegger, Petr Procházka

**Affiliations:** 1 Institute of Vertebrate Biology, Academy of Sciences of the Czech Republic, v.v.i., Květná 8, CZ-60365 Brno, Czech Republic; 2 Department of Bird Migration, Swiss Ornithological Institute, Seerose 1, CH-6204 Sempach, Switzerland

**Keywords:** geolocator, habitat deterioration, NDVI, passerines, sub-Saharan Africa, vegetation conditions

## Abstract

Migratory birds often move significantly within their non-breeding range before returning to breed. It remains unresolved under which circumstances individuals relocate, whether movement patterns are consistent between populations and to what degree the individuals benefit from the intra-tropical movement (ITM). We tracked adult great reed warblers *Acrocephalus arundinaceus* from a central and a southeastern European breeding population, which either stay at a single non-breeding site, or show ITM, i.e. move to a second site. We related ITM to the normalized difference vegetation index (NDVI) describing vegetation conditions and probably reflecting food abundance for these insectivorous birds. Three-quarters of birds showed ITM across the non-breeding range. We found no difference in range values and mean values of NDVI between the single non-breeding sites of stationary birds and the two sites of moving birds. The vegetation conditions were better at the second sites compared to the first sites during the period which moving birds spent at the first sites. Vegetation conditions further deteriorated at the first sites during the period the moving birds resided at their second sites. Our study provides evidence that birds probably benefit from improved conditions after ITM compared to the conditions at the sites from where they departed.

## 1. Introduction

A large number of bird species across the globe annually undertake long-distance migration [1,2]. After arriving at their non-breeding grounds, many of these species show additional intra-tropical movements within the non-breeding areas [3–5]. This phenomenon is generally known as itinerancy [6–8] and occurs in both the Nearctic-Neotropical [9–11] and Palearctic-African migration system [12–15]. Intra-tropical movements (hereafter ITMs) result in successive occurrence in at least two non-breeding sites situated in the tropical zone [5]. Further, ITMs differ from directional intercontinental migration by the absence of returning to the starting location. Simultaneously, relocation across long distances (up to thousands of kilometres) following a long residence period at the first non-breeding site [5] does not fit short-distance displacements [16]. Although ITMs are assumed to be important for population dynamics [5], detailed tracking data in reasonable sample sizes have been unavailable until recently and, thus, the link between the ITMs and changes in environmental conditions was rarely investigated.

In the Palearctic-African migration system, birds usually arrive in tropical non-breeding areas during the rainy season from August to October [17]. There, at constantly high temperatures, rainfall promotes the development of vegetation and, consequently, determines the abundance of herbivorous invertebrates [18–20]. In the Sahel zone—a major non-breeding region for many insectivorous passerine species [21]—insect availability decreases when the Sahel gradually dries up from November to May. In some migrants, this period coincides well with ITM that enables the birds to move from the first non-breeding site with decreasing food availability to another non-breeding site [7,21,22]. Yet, the consistency and benefits of this ITM in Africa are still insufficiently understood. Namely, it remains unresolved (i) under which conditions individuals leave or stay at the first site, (ii) if these patterns are consistent in different populations and (iii) to which degree the individuals benefit from ITM.

We studied ITM in the great reed warbler (*Acrocephalus arundinaceus*), a long-distance migratory passerine species. Its broad non-breeding distribution covers major parts of sub-Saharan Africa from Senegal to South Africa [23], where it occupies a wide array of wet as well as dry habitats including crop fields (summarized by Leisler [24]). After crossing the Sahara in August and September, great reed warblers stay in the Sahel zone usually until November or December, probably to undergo complete moult [25], and then some move to second (final) non-breeding sites often situated further south (hereafter ‘movers’ [4,12]) or stay until the departure from Africa which takes place mostly in April (hereafter ‘non-movers’). We here related non-breeding site occupancy derived from light-level geolocation to corresponding vegetation conditions by using remotely sensed vegetation data. Insect abundance is supposed to be positively related to primary productivity: greener vegetation may imply a higher abundance of insects [26–28] and thus more favourable conditions for insectivorous birds. To estimate plant productivity at individual non-breeding sites, we used the normalized difference vegetation index (hereafter NDVI [29–31]).

Non-movers have a single non-breeding site (hereafter ‘single site’) and cope with the environmental conditions at one site over the whole non-breeding period—i.e. including the beneficial rains as well as the adverse dry period. Thus, we predict that (i) the overall seasonal range of the vegetation conditions experienced by non-movers is expected to be wider than that experienced at the two non-breeding sites by movers which should face rather favourable conditions throughout the non-breeding period. If (ii) the habitat suitability at the first non-breeding site of movers (hereafter ‘first site’) is lower than at their second non-breeding site (hereafter ‘second site’), it is beneficial to move from the first to the second site. Simultaneously, (iii) vegetation conditions at the second site are expected to be better than at the first site during the presence at the second site. Finally, we predict that (iv) the deterioration of vegetation conditions before leaving the first site will be stronger than at the single site of non-movers.

## 2. Material and methods

### 2.1. Geolocator application and analysis

Geolocators were attached to adult great reed warblers of a central European (hereafter ‘central’) population breeding in southeastern Czech Republic (48°53′ N, 17°3′ E) and of a southeastern European (hereafter ‘southeastern’) population in northeastern Bulgaria (44°0′ N, 26°26′ E). The central population spends the non-breeding period in a relatively narrow belt in western sub-Saharan Africa [4] latitudinally restricted from the south by the Gulf of Guinea. The southeastern population, on the contrary, occupies large grounds in central Africa, which are considerably less restricted in terms of geography, because the birds may undertake longer southbound movements towards their second sites [4].

Birds were captured during the breeding seasons 2012–2015 and equipped with geolocators (0.72–1.36 g, 7 mm light guide), type SOI-GDL1.0, SOI-GDL3.0 PAM (Swiss Ornithological Institute) and Intigeo-P65B1-7 (Migrate Technology; www.migratetech.co.uk) mounted with silicone, neoprene or nylon leg-loop harness (see electronic supplementary material, table S1 for details). In central Europe, we obtained geolocator data from 19 of 129 males and 10 of 94 females, and in southeastern Europe, we obtained tracking data for 11 of 73 males and 6 of 29 females. An additional 45 birds had lost the logger or the device contained insufficient data, and a total return rate across the two populations reached 28.0% (for details see electronic supplementary material, table S1). In further analyses, we did not compare sexes due to a low number of males or females in some classes—e.g. only one male non-mover and three female movers in the southeastern population.

We applied the threshold method to determine sunrise and sunset times for each recorded day [32]. To obtain an appropriate sun elevation angle for the used habitat, each geolocator was calibrated on the bird at the breeding site, during the post-breeding and (if available) pre-breeding period (in-habitat calibration [33]). The resulting angles varied between –6.5° and 3.1° depending on the type of geolocator, habitat and individual bird behaviour. Stationary periods and geographical positions were determined using GeoLight 1.03 [34] in R [35], following the procedure given in Emmenegger *et al.* [31]. We excluded sun events outside two interquartile ranges (*k*) using the loessFilter function. Stationary periods were determined using the changeLight function with a threshold at the 0.9 quantile of change point probability and a 3-day minimum staging time. We merged consecutive stationary periods when the daily positions markedly overlapped and the average positions of the non-breeding sites (see below) were not farther than approximately 200 km [4]. Those which were farther were considered as separate non-breeding sites. We used an average of the sun elevation angles obtained from on-bird calibration for sub-Saharan non-breeding sites of the particular bird [4].

We defined the position of each non-breeding site as the peak of the frequency distributions (mode) of both latitudes and longitudes of the daily positions. In line with Koleček *et al.* [4], individual non-breeding sites were located within sub-Saharan Africa (south of 20° N) and were occupied in the period between 1 September and 31 March.

### 2.2. Spatial and temporal pattern of intra-tropical movements

**Figure 1.**
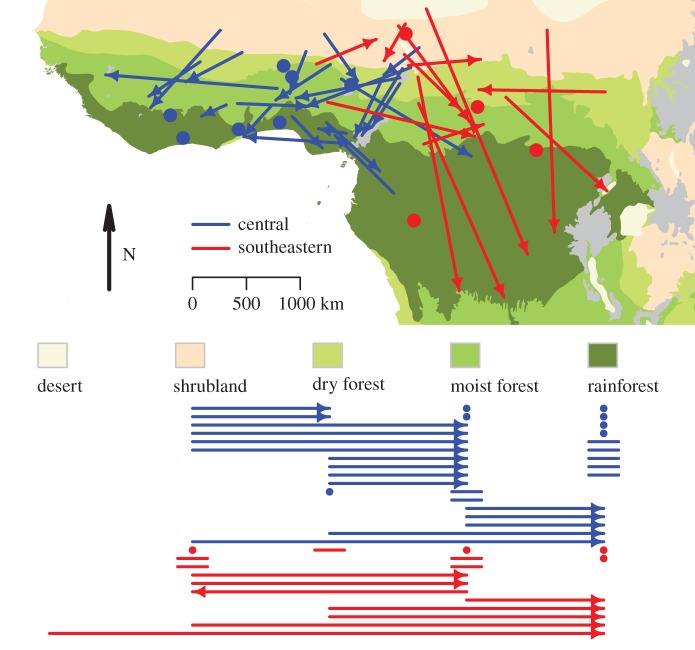
Movements between the first and the second sub-Saharan non-breeding sites of 22 birds from the central European population and 13 birds from the southeastern European population with two non-breeding sites. Arrows connect individual locations and denote the movement directions of the movers. Each site is expressed as the centre of the highest density distribution of daily positions within a stationary period (mode). Positions of seven non-movers from the central and four birds from the southeastern European population are depicted with filled circles. See electronic supplementary material, figure S1 for the 1st and 3rd quartiles in longitude and latitude of individual positions. Presence in individual ecological zones [36] is summarized below the legend: movements between the zones, arrows; movements within a zone, lines; residency, filled circles. One arrow/line/circle represents one bird. Grey colour denotes mountains.

We defined ITM as the movement between two non-breeding sites of movers. To explore to what extent the movers change non-breeding sites in relation to changes in habitat conditions at a large spatial scale (i.e. from rapidly drying-up Sahel to rainforest without such marked seasonality), we calculated the proportion of movers that flew from a drier to a moister ecological zone defined along a gradient of habitat wetness and increasing complexity of vegetation structure: (i) deserts, (ii) shrubland, (iii) dry forest, (iv) moist forest, and (v) rainforest ([36], figure 1).

We determined loxodromic distances (km) between the first and the second site using the ‘birdring’ package [37] and the directions of ITM using Oriana 3.21 [38]. We tested whether a mean movement direction of the focal populations differs from a random distribution [39] and whether distance and direction of ITM differ between the two populations.

### 2.3. Vegetation phenology of non-breeding sites

We used NDVI time series to characterize temporal changes in primary productivity at the sub-Saharan non-breeding sites, wherein higher NDVI values give high photosynthetic activity and thus greener vegetation [30]. Usually, NDVI correlates with precipitation (e.g. [40,41]) and thus also indirectly reflects humidity in a focal area. The weekly NDVI data were available in a 16 × 16 km grid at ftp://ftp.star.nesdis.noaa.gov/pub/corp/scsb/wguo/data/VHP_16km/. As the accuracy of geolocation is rather limited [33], mean weekly NDVI at each non-breeding site was calculated from 3 × 3 cells (i.e. 48 × 48 km), where the central cell included the estimated position of the site. We extracted two sets of weekly NDVI values—namely, (i) for the period when an individual was present at the non-breeding site (i.e. single site of non-movers and first and second site of movers) as well as (ii) for the period when the mover was present at the other of the two sites. Subsequently, we calculated the mean NDVI for each non-breeding site and period.

### 2.4. Intra-tropical movement and vegetation phenology

We distinguished among (i) the single site of non-movers, (ii) the first site during the period when a mover was present, (iii) the second site during the period when a mover was present, (iv) the second site during the period which a mover spent at the first site and, conversely, (v) the first site during the period which a mover spent at the second site. We tested whether the mean NDVI differed between non-breeding sites when the movers were present and when they stayed at the other non-breeding site. We applied linear mixed-effect models (R package lme4, [42]) with the site-specific mean NDVI (at the sites i–v) as a response variable and their location (single site of non-movers, first or second site of movers) nested in the bird ID nested in the study year as random effects. ‘Location’ accounted for the fact that we obtained two NDVI values (i.e. when the mover was present or absent) from the first (sites ii and v) and second non-breeding location (sites iii and iv), and that these were not independent in space. Bird ID accounted for the differences between individuals and study year for potential annual differences. We fitted the model with a two-way interaction between non-breeding site and population to explore the population differences.

We compared the individuals' ranges of weekly NDVI at their non-breeding sites to test whether the non-movers experienced a larger variability of NDVI than the movers. To identify the relation between the timing of ITM and the site-specific vegetation phenology, we calculated (i) the change in weekly NDVI (*Δ*NDVI) during the two weeks around arrival at and around departure from the first or the second site and (ii) *Δ*NDVI between the first and the second site during the two weeks around movement. We fitted a linear mixed-effect model with *Δ*NDVI as a response variable, a two-way interaction between the category of *Δ*NDVI and population as predictors, and bird ID nested in the year of geolocator attachment as a random term. We also tested whether the vegetation conditions deteriorated more strongly at the first sites of movers before departure or at single sites of non-movers at the same time. At the first sites, where the weekly NDVI declined before departure (*N* = 19 for the central, 10 for the southeastern population), we calculated the slope of the decline using linear regression. At the single sites of non-movers (*N* = 7 in central and 4 in southeastern population), we repeated this exercise over a similar, i.e. up to nine-week period (i.e. average length of declining NDVI before the departure from first sites in movers) after NDVI had started to decline. Further, we compared the slopes between the decline at first (movers) and single sites (non-movers) using a generalized linear mixed-effect model with binomial error distribution and logit link function [42]. The decision whether to stay (0) or move to the second site (1) was entered as a response variable, and we controlled for latitude of the respective sites and also took study year into account as a random factor. Finally, we related the *Δ*NDVI between the first and second site to the distance between these sites.

## 3. Results

### 3.1. Spatial and temporal pattern of intra-tropical movements

ITM occurred in 76% of birds similarly in the central and the southeastern population (*χ^2^* = 0.002, *p* = 0.963). Out of 35 movers, 68% of birds from the central and 54% of birds from the southeastern population switched from a drier to a moister ecological zone (difference between populations: *χ^2^* = 0.70, *p* = 0.403). The remaining individuals moved within the same ecological zone, with one exception which moved from a moister to a drier zone in the southeastern population (figure 1).

**Figure 2.**
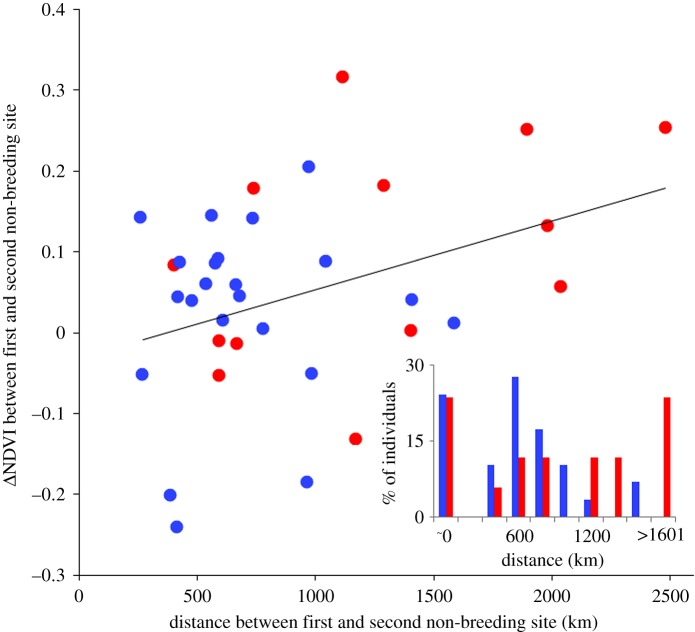
Positive relationship between changes in NDVI upon departure from the first and the arrival at the second non-breeding site and the covered distance. Black line denotes the regression. An inset bar plot shows frequency distribution of movement distances with the southeastern European population (red) moving further than the central European population (blue).

The distance of ITM ranged between 251 and 1581 km (median = 593 km) in the central population (figures 1 and 2), being significantly shorter than the 394–2476 km (median = 1163 km) in the southeastern population (Mann–Whitney–Wilcoxon test: *W* = 221, *p* = 0.007, figures 1 and 2).

The average direction of ITM in both populations differed significantly from a random direction and from each other (Watson–Williams *F*-test: *F*_1,33_ = 15.83, *p* < 0.001). In the central population birds moved towards the southwest (mean vector = 222.6°; *r* = 0.62; Rayleigh *Z* = 8.32, *p* < 0.001, *N* = 22, figure 1) and in the southeastern population towards the southeast (mean vector = 139.5°; *r* = 0.64; Rayleigh *Z* = 5.30, *p* = 0.003, *N* = 13, figure 1).

ITM in the central population started between 20 October and 15 February (median = 25 November) and lasted between 1 and 10 days (median = 2). In the southeastern population, ITM started between 11 November and 12 January (median = 4 December) and lasted 1–9 days (median = 2). The date of departure from the first site did not differ significantly between years (*R^2^* = 0.05; *F*_2,32_ = 0.83; *p* = 0.444).

### 3.2. Vegetation phenology

**Figure 3.**
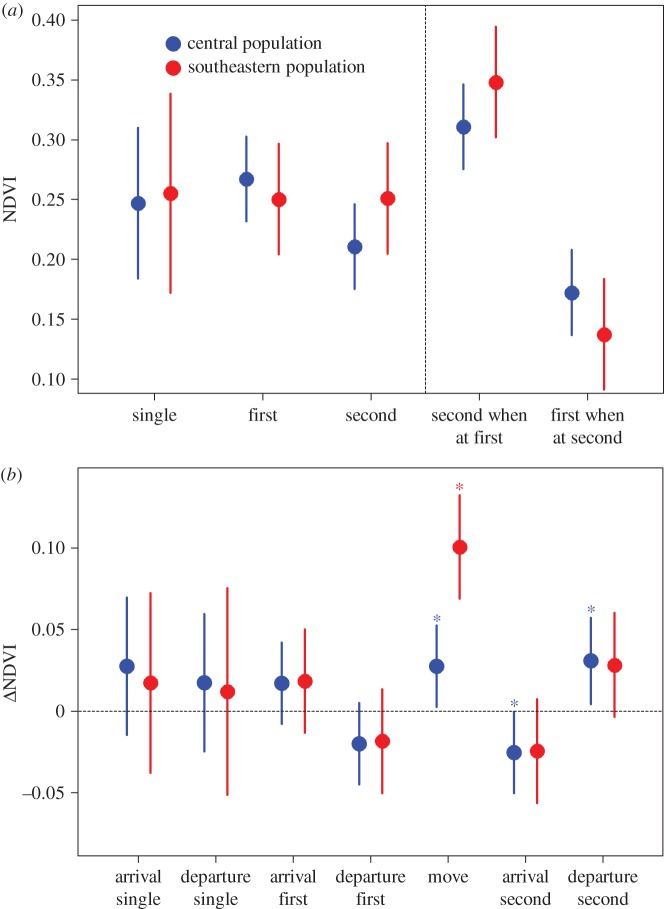
(*a*) Mean NDVI at the first and the second non-breeding sites of movers as well as at the first sites during the period spent at the second sites (first when at second) and vice versa (second when at first) and at the single sites of non-movers. (*b*) *Δ*NDVI between one week before and one week after arrival/departure at/from individual sites and between the departure from the first and arrival at the second site (‘move’; *N* = 29 birds from central and 17 from southeastern European population). Least-square means (95% CI) estimated by the model containing the interaction effects of population and (*a*) non-breeding site/(*b*) arrivals/departures from the sites are shown. Asterisks indicate significance of difference from zero (dashed line).

When the mean NDVI at the first and second sites of movers were averaged (0.238 ± 0.067 s.e.), they did not differ from the mean NDVI at single sites of non-movers in both populations (two-sample *t*-test: 0.250 ± 0.087 s.e., *t* = –0.50, *p* = 0.622, figure 3*a*). Similarly, the mean range of weekly NDVI did not differ (single site: 0.173 ± 0.024 s.e.; two sites: 0.214 ± 0.012 s.e., two-sample *t*-test: *t* = –1.58, *p* = 0.675). For the central population, the NDVI of the first site when occupied was higher than at the second site when occupied (estimate: –0.057 ± 0.024 s.e., *p* = 0.022, figure 3*a*); NDVI of the first and second sites when occupied did not differ in the southeastern population (estimate: 0.001 ± 0.031 s.e., *p* = 0.982, figure 3*a*).

When present at the first site, the mean NDVI at the still unoccupied second sites of the southeastern population was significantly higher compared to the first sites (estimate: 0.098 ± 0.031 s.e., *p* = 0.003, figure 3*a*), and this relationship was marginally insignificant for the central population (estimate: 0.044 ± 0.024 s.e., *p* = 0.075, figure 3*a*). Later in the season, when the movers resided at the second non-breeding site, NDVI at the first sites became drastically lower than at the first (central population: –0.095 ± 0.016 s.e., *p *< 0.001; southeastern population: –0.113 ± 0.021 s.e., *p *< 0.001, figure 3*a*) and, namely in the southeastern population, even the second sites when occupied (central population: –0.038 ± 0.024 s.e.; *p* = 0.118; southeastern population: –0.011 ± 0.031 s.e., *p* = 0.001, figure 3*a*). NDVI at individual sites did not differ between years (*R^2^* = 0.01; *F*_2,148_ = 0.45; *p* = 0.636).

*Δ*NDVI around arrival/departure from non-breeding sites did not differ significantly from 0 at most sites in both populations (figure 3*b*). Moreover, changes in NDVI when moving from the first to the second site were less pronounced in the central compared to the southeastern population (estimate: –0.073 ± 0.019 s.e., *p *< 0.001, figure 3*b*). The weekly NDVI did not decline more steeply at first sites of movers before departure than at the single sites of non-movers at the same time (estimates for the central population: –0.800 ± 0.615 s.e., *p* = 0.193; for the southeastern population: –0.208 ± 1.300 s.e., *p* = 0.110) and the latitude of first and single sites did not differ either (estimates for the central population: 0.971 ± 0.605 s.e., *p* = 0.108; for the southeastern population: 1.543 ± 1.208 s.e., *p* = 0.201). Finally, *Δ*NDVI during ITM was positively related to ITM distance (slope = 0.05, *R^2^* = 0.13, *F_1,33_* = 4.98, *p* = 0.033, figure 2).

## 4. Discussion

Our study shows that great reed warblers undertaking ITMs occupy sites with improved conditions after movements compared to the conditions at the original site. ITMs occurred at similar frequencies in both populations, but were longer in birds from the southeastern population spending their non-breeding period in central Africa than in birds from the central population dwelling in Western Africa, suggesting that their ITM towards southern areas is limited by the Gulf of Guinea [4].

Most ITMs were recorded from drier to wetter ecological zones with the onset of the dry period. However, a quarter of the population did not move and another quarter moved within the same ecological zone. This implies that ITM is an optional strategy, probably an adaptive response to fluctuating environmental conditions [43], and contrasts with the long-distance latitudinal migration that is obligatory for all birds at any conditions [1].

Unlike the post- and pre-breeding migrations, which are usually not varying more than several weeks between individuals [4], ITM occurred over a broad period of several months, i.e. from October to February. Such a timespan may suggest some plasticity in the behavioural responses to changes in the environment. The wide individual and within-population variation in the timing of ITM also points towards significant variability in local habitat conditions encountered by the study species. Specifically, the natural water regime and drying-out of the local habitat is decisive for a wide array of passerine migrants [21,44]. On the one hand, for some great reed warblers, larger freshwater bodies will dry out more slowly and may provide favourable conditions for a longer time than smaller wetlands. On the other hand, other individuals may avoid drying-out sites and leave or occupy drier habitats [24], which, however, respond more quickly to weather changes and force them to leave early. Together with the temporal variability of ITM, the absence of marked changes in vegetation conditions around arrival on or departure from non-breeding sites suggests that the birds' response to the gradually changing environmental conditions is highly individual.

Our study inevitably combined conditions inferred with higher uncertainty, i.e. exact positioning and vegetation as well as foraging conditions, with rather precise data such as timing and proportion of ITM, though when averaging across populations, the inaccuracy should be relatively lower than at the individual level. Geolocation by light does not allow for positioning with very high accuracy [33] and thus hampers any evaluation of local, site-specific habitat conditions. Investigation of the variability of exploited habitats at individual non-breeding sites of most small body-sized migrants would therefore require other approaches. Nevertheless, local habitat changes generally result from the wider conditions at intermediate spatial scales and thus we are confident that we were able to track relevant patterns.

The vegetation conditions changed more dramatically between the first and the second site when these were more distant, especially in the southeastern population. This result follows the differences in vegetation conditions between ecological zones [36], which are, naturally, larger across larger distances, i.e. rather in Eastern than in Western Africa. Regardless of the distance, movements from the first to the second site were fast as most movers switched their location within just 2 days (max. 10 days). This indicates that they moved to specific areas without lengthy search for suitable sites, building on experience from previous years [45].

Both populations experienced similar vegetation conditions, but movers from the central population faced more severe habitat deterioration at the second sites, probably due to the onset of the dry period in West Africa [43]. However, the birds from both populations would have experienced even worse conditions if they had stayed at their first sites. Interestingly, vegetation conditions were similar at the first and second sites of movers from the southeastern population, which frequently chose their second sites in southern parts of the non-breeding range. These birds arrived at their second sites with better habitat conditions than at their previous sites, because at the second sites the rainy season has ended only recently [46]. Afterwards, however, even these sites started to dry up and, therefore, the ‘average’ conditions here were similar to those at the first sites. Despite this benefit, movers from the southeastern population might compete with a wider range of tropical resident species or might be more limited by increased travel costs [47] than movers from the central population. Indeed, the absence of droughts at the southernmost sites before leaving Africa offers potentially better conditions to prepare for longer pre-breeding migrations as found in the Neotropics [48].

Palearctic migrants generally prefer areas with large seasonal resources during the wet season [49], such as the Sahel zone from August to October [17]. If the migrants flew directly to moister southern areas, they would possibly face a stronger competition by tropical resident species (see also [50]). As a potential consequence, moult as an energy-demanding period of the annual cycle should be accomplished at northern sites, which seems to be the case in great reed warblers [1,7,51,52]. Besides that, individuals arriving in tropical Africa are expected to be exhausted after crossing the Mediterranean Sea and the Sahara due to limited possibilities for refuelling [4,45] and thus it seems beneficial to stop in the closest suitable areas—i.e. in the Sahel during the rainy season.

Unexpectedly, the absolute values of vegetation conditions, their range and their deterioration after the onset of the dry period faced by non-movers were similar to those in movers. Moreover, the vegetation conditions at single sites of non-movers were similar to conditions at the first movers' sites until the end of the non-breeding period (two-sample *t*-test: 0.192 ± 0.014 s.e., *t* = 0.65, *p* = 0.518), indicating that non-movers did not choose sites with more stable conditions than movers. Studds & Marra [53] reported that wet habitat of high quality (mangroves) can suppress the negative effect of the dry season even if the perturbation lasted several months. This might also be the case of some sub-Saharan sites (e.g. large lakes or water reservoirs), which probably keep suitable conditions for a part of Sahel-dwelling migrants, thus enabling them to spend the whole stationary non-breeding period at such sites.

In brief, we demonstrated that switching between tropical non-breeding areas within a given season results in more favourable vegetation conditions compared to those at a single site. Birds could theoretically also skip the first non-breeding grounds in the Sahel and fly directly to the second non-breeding sites, but they might face competition with tropical residents or limited fuel on post-breeding migration. Future research should thus combine tracking habitat changes at a finer spatial scale with ITM and individual performance like recovery after post-breeding migration, moult or refuelling before departure from Africa.

## Supplementary Material

Details on tracked birds and geolocators

## Supplementary Material

Nonbreeding sites of the tracked great reed warblers with the quartiles in longitude and latitude

## Supplementary Material

R code used for analyses and explanatory notes

## Supplementary Material

Data used for the analysis of difference in mean NDVI between nonbreeding sites

## Supplementary Material

Data used for the analysis of change in weekly NDVI around arrival and departure

## Data accessibility

The data and codes underlying analyses are available as electronic supplementary material, S1–S3.
