# Supplementary material for: Intra-tropical movements as a beneficial strategy for Palearctic migratory birds
Source: R Soc Open Sci. 2018 Jan 3;5(1):171675. doi: 10.1098/rsos.171675 (PMC5792944; doi:10.1098/rsos.171675)
Supplement: Details on tracked birds and geolocators [file rsos171675supp1.doc]

**ELECTRONIC SUPPLEMENTARY MATERIAL**

**Koleček J, Hahn S, Emmenegger T, Procházka P. 2017 Intra-tropical movements as a beneficial strategy for Palearctic migratory birds. R. Soc. Open Sci. 4: 171675.**

**Table S1.** Capture year and number of captured and recaptured (with data for this paper / all recaptured) individuals, and technical details of geolocators (type, harness material, angle of light guide and mass [g]) used to track great reed warblers.

| Capture year | Deployed | Recaptured | Type | Harness material | Angle | Mass |
| --- | --- | --- | --- | --- | --- | --- |
| *Central Europe* | |  |  |  |  |  |
| 2012 | 16 M, 16 F | 4/8 M, 4/4 F | SOI-GDL1.0 | silicone | 45–60° | 1.22 |
| 2013 | 33 M, 38 F | 0/12 M, 0/13 F | SOI-GDL1.0 | neoprene | 45–60° | 1.22 |
| 2014 | 46 M, 24 F | 6/13 M, 2/8 F | SOI-GDL1.0 | nylon | 45–60° | 1.22 |
| 2015 | 34 M, 16 F | 9/9 M, 4/4 F | Intigeo-P65B1-7 | nylon | 45° | 0.72 |
| *Southeastern Europe* | |  |  |  |  |  |
| 2012 | 22 M, 10 F | 4/5 M, 1/1 F | SOI-GDL1.0 | silicone | 45–60° | 1.23 |
| 2015 | 51 M, 19 F | 7/7 M, 5/7 F | SOI-GDL3.0 PAM | nylon | 45–60° | 1.36 |
| ***Both populations in total*** | |  |  |  |  |  |
| **2012–2015** | **202 M, 123 F** | **30/54 M, 16/37 F** |  |  |  |  |
