# Supplementary material for: Intra-tropical movements as a beneficial strategy for Palearctic migratory birds
Source: R Soc Open Sci. 2018 Jan 3;5(1):171675. doi: 10.1098/rsos.171675 (PMC5792944; doi:10.1098/rsos.171675)
Supplement: Nonbreeding sites of the tracked great reed warblers with the quartiles in longitude and latitude [file rsos171675supp2.doc]

**ELECTRONIC SUPPLEMENTARY MATERIAL**

**Koleček J, Hahn S, Emmenegger T, Procházka P. 2017 Intra-tropical movements as a beneficial strategy for Palearctic migratory birds. R. Soc. Open Sci. 4: 171675.**

**Figure S1.** Non-breeding sites of 29 great reed warblers from central European population and 17 birds from southeastern European population. Individual positions are expressed as the centres of the highest density distributions of daily positions (modes; filled circles) and the 1st and 3rd quartiles in longitude and latitude (lengths of crossing vertical and horizontal bars).
